# Au-siRNA@ aptamer nanocages as a high-efficiency drug and gene delivery system for targeted lung cancer therapy

**DOI:** 10.1186/s12951-020-00759-3

**Published:** 2021-02-24

**Authors:** Yuming Yang, Yu Han, Qiuyang Sun, Jin Cheng, Caixia Yue, Yanlei Liu, Jie Song, Weilin Jin, Xianting Ding, Jesús M. de la Fuente, Jian Ni, Xiaoqiang Wang, Daxiang Cui

**Affiliations:** 1 Institute of Nano Biomedicine and Engineering, Key Laboratory for Thin Film and Microfabrication Technology of the Ministry of Education, Shanghai Engineering Research Center for Intelligent Diagnosis and Treatment Instrument, Department of Instrument Science & Engineering, School of Electronic Information and Electrical Engineering, Shanghai Jiao Tong University, 800 Dongchuan Road, Shanghai, 200240 People’s Republic of China; 2 National Center for Translational Medicine, Collaborative Innovational Center for System Biology, Shanghai Jiao Tong University, 800 Dongchuan RD, Shanghai, 200240 People’s Republic of China; 3 Pediatric Neurological Disease Center, Xinhua Hospital, Shanghai Jiaotong University School of Medicine, Number 1665, Kongjiang Road, Shanghai, 200092 People’s Republic of China; 4 School of Biomedical Engineering, Shanghai Jiao Tong University, 200240 Shanghai, People’s Republic of China; 5 Instituto de Nanociencia de Aragon (INA), Universidad de Zaragoza, Zaragoza, 50018 Spain

**Keywords:** Lung cancer, Gold nanoparticle, Gene delivery, Tumor targeted therapy, Gold nanocage

## Abstract

**Background:**

Gene and chemical therapy has become one of the rising stars in the field of molecular medicine during the last two decades. However, there are still numerous challenges in the development of efficient, targeted, and safe delivery systems that can avoid siRNA degradation and reduce the toxicity and adverse effects of chemotherapy medicine.

**Results:**

In this paper, a highly efficient AS1411 aptamer modified, dsDNA and MMP-2 cleavable peptide-fabricated gold nanocage vehicle, which could load doxorubicin hydrochloride (DOX) and siRNAs to achieve a combination of tumor responsive genetic therapy, chemotherapy, and photothermal treatment is presented. Our results show that this combined treatment achieved targeted gene silencing and tumor inhibition. After nearly one month of treatment with DOX-loaded Au-siRNA-PAA-AS1411 nanoparticles with one dose every three days in mice, a synergistic effect promoting the eradication of long-lived tumors was observed along with an increased survival rate of mice. The combined genetic, chemotherapeutic, and photothermal treatment group exhibited more than 90% tumor inhibition ratio (tumor signal) and a ~ 67% survival rate compared with a 30% tumor inhibition ratio and a 0% survival rate in the passive genetic treatment group.

**Conclusions:**

The development of nanocarriers with double-stranded DNA and MMP-2 cleavable peptides provides a new strategy for the combined delivery of gene and chemotherapy medicine. Au-siRNA-PAA-AS1411 exerts high anticancer activities on lung cancer, indicating immense potentials for clinical application.
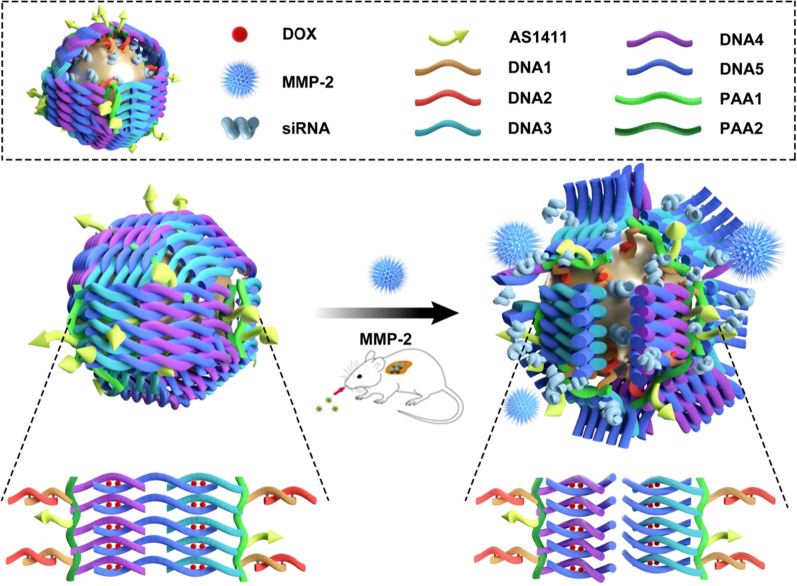

## Background

Lung cancer is one of the most common malignant tumors in the world. The five-year survival rate of patients with lung cancer remains very low due to difficulties of early diagnosis, drug resistance, and relapse after treatment. So far, surgical treatment is considered to be the only radical curative method, but most patients have reached the advanced stage by the time they seek treatment, and thus the optimal window for radical surgery or radiotherapy is lost. New strategies are needed to improve the tolerability and efficacy of treatment for lung cancer. Gene therapy is an efficient and promising approach to treating malignant tumors by either degrading mRNAs or inhibiting their translation. Many siRNAs have been shown to be actively involved in metabolism, cell death, angiogenesis, metastasis and immunosuppression in cancer [1]. Using siRNAs as therapeutic agents for regulating cancer can alter the intrinsic properties of tumor cells and the tumor microenvironment. Increasing attention has been focused on gene therapy due to its potential use in treating a variety of diseases, including cancers, by delivering genetic drugs to the target tumor sites [2–4]. Among various genetic drugs, the vascular endothelial growth factor (VEGF) family (VEGF A, B, C, D and Placenta growth factor) has been the most widely investigated [4–9]. The development of VEGF-targeted genetic drugs, including VEGF-A receptors, small molecule tyrosine kinase inhibitors and antibodies, has been widely investigated, and these drugs have been confirmed to be efficient anti-angiogenesis therapies [10–12]. However, naked siRNAs are difficult to use for in vivo applications, as they are easily degraded by serum nucleases and are rapidly eliminated through renal excretion. Also, to efficiently deliver to target sites, siRNAs should be considered in sufficient numbers. Therefore, a suitable gene delivery system that can protect nucleic acids from degradation and selectively deliver a sufficient amount of siRNAs to target sites is needed.

Nanoparticles (NPs) are effective carriers of multiple therapeutic and diagnostic agents [13–17]. Various methods have been reported for the modification of nanoparticles to confer the NPs good bioavailability, low toxicity and a long circulation time [18–21]. NPs are now widely applied in oncology research for tumor imaging and theranostics [22–24]. NPs used for siRNA delivery have also been reported [25]. Chen et al. reported a magnetic mesoporous silica nanoparticle (M-MSN)-based siRNA delivery system for the delivery of VEGF-small interfering RNA (siRNA) [5]. Ding et al. reported polyethyleneimine (PEI)-modified single-walled carbon nanotubes to deliver VEGF-targeted siRNA (siVEGF) for the synergistic and targeted treatment of tumor angiogenesis [26]. James Finlay et al. synthesized polyethyleneimine-coated mesoporous silica nanoparticles (MSNs) for the delivery of siRNA, which led to a reduction in Twist1 [27]. However, these systems cannot protect the siRNA with a closed environment to avoid degradation. The amount of siRNAs delivered is also limited compared with the ratio of gene carries. In addition, a good vehicle should also have the ability to selectively transport oligonucleic acid-based therapeutics to the target sites and execute the controlled release the gene to the diseased tissue.

In the current study, a nanocage was designed for the co-delivery of gene and chemotherapy drugs. Au nanoparticles were selected as the vesicles due to their many promising characteristics, including controllable sizes and shapes, biocompatibility, nontoxicity, and ease of connecting with the target molecules due to the strong interactions with amine or thiol moieties. The distinguished X-ray attenuation property of Au nanoparticles enables these particles to serve as one of the most effective contrast agents for X-ray CT imaging. Besides, Au nanoparticles can absorb near-infrared laser frequencies to achieve thermal ablation of cancer cells [28–31]. Polyacrylic acid (PAA), a popularly used material, was used in this study to construct the surface of the cage by taking advantage of the numerous carboxylic acid groups available for biomolecule conjugation [32–35]. The strongly negative surface charge of PAA also conferred the Au nanocage good biocompatibility and water dispersity. Double-stranded DNA (dsDNA) molecules act as the rigid trestle in the gold nanocage structure. One single-stranded DNA molecule, ssDNA-2, was modified with thiol groups and anchored with the Au nanoparticle, and the complementary antisense strand, ssDNA-1, was modified with NH_2_ and connected with PAA through an amidation reaction. In addition, PAA was divided into two groups and modified with two kinds of complementary amino-modified oligonucleotides (ssDNA-3, ssDNA-4) and AS1411. ssDNA-2 was first anchored to the Au nanoparticle surface by the Au–S bond, and then the PAAs were modified with the complementary antisense oligonucleotide, ssDNA-1, which was hybridized to ssDNA-2. The gold nanocages were closed through the complementary base pairing of DNA-3 and DNA-4 with DNA-5 (linked with the MMP-2 cleavable peptide). Then, a large number of dsDNA molecules and peptides were stacked on the surface of the Au nanocages, rendering the Au nanoparticles as closed nanocages. To further enhance the antitumor efficacy, doxorubicin was introduced into this nanocage system by taking advantage of the property that DOX can preferentially intercalate into double-stranded 5′-GC-3′ or 5′-CG-3′ to form a tightly coupled complex without chemical bond links [36]. The gold nanocages could selectively deliver the loading drugs and genes to the tumor sites due to modification of AS1411, anti-nucleolin aptamers for site-specific targeting against tumor cells, which overexpresses nucleolin receptors. The nanocages were destroyed by the matrix metalloproteinase (MMP)-2 enzymes, which were overexpressed around the tumor tissues by the cleavage of the enzyme-cleavable peptide chain (contained in DNA-5) on the surface of the gold nanocages. Following the cleavage of the peptide chain, the tumor-targeted and controlled drug release was achieved.

**Scheme 1:**
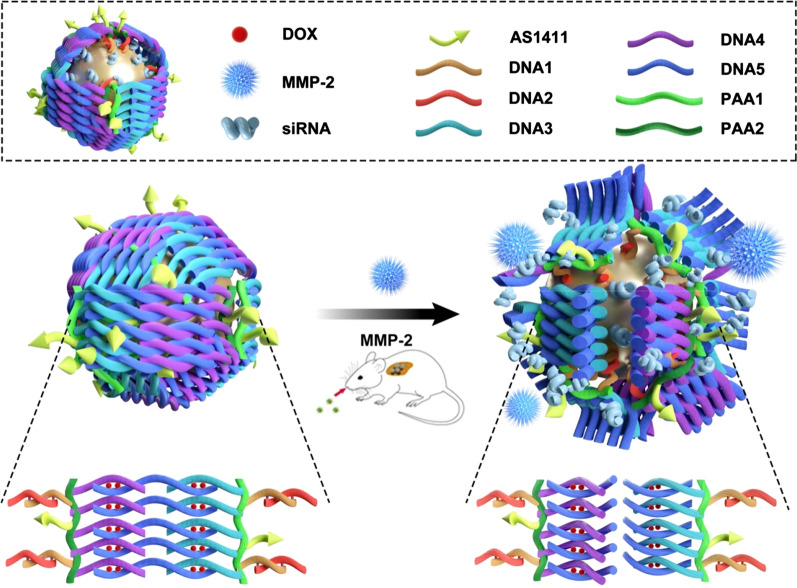
The schematic illustration of the construction of gold nanocages and the tumor-induced gene- and DOX-releasing mechanism. Gold nanoparticles were first decorated with thiolated-DNA-2 and thiolated anti-VEGF siRNAs based on the thiol-Au bond. PAA-1 was modified with ssDNA-1, ssDNA-3, and the AS1411 aptamer. PAA-2 was modified with ssDNA-1, ssDNA-4, and the AS1411 aptamer; then, the ssDNA-2 and siRNA-conjugated (DNA-2: siRNA = 1:10 molar ratio) gold nanoparticles were conjugated with equal molar ratios of PAA-1, PAA-2, and DNA-5 to generate the rigid trestle of the gold nanocages. The gold nanocage were finally closed through the complementary base pairing of DNA-3 and DNA-4 with DNA-5

To our knowledge, this is the first proof of concept to construct a tumor responsive drug delivery nanocage system using Au nanoparticle, dsDNA, and MMP-2 cleavable peptide. The developed gold nanocage system shows good stability, excellent tumor inhibition ratio, and high gene delivery efficacy (Scheme 1).

## Results and discussion

### Au nanocage design and characterization

**Fig. 1:**
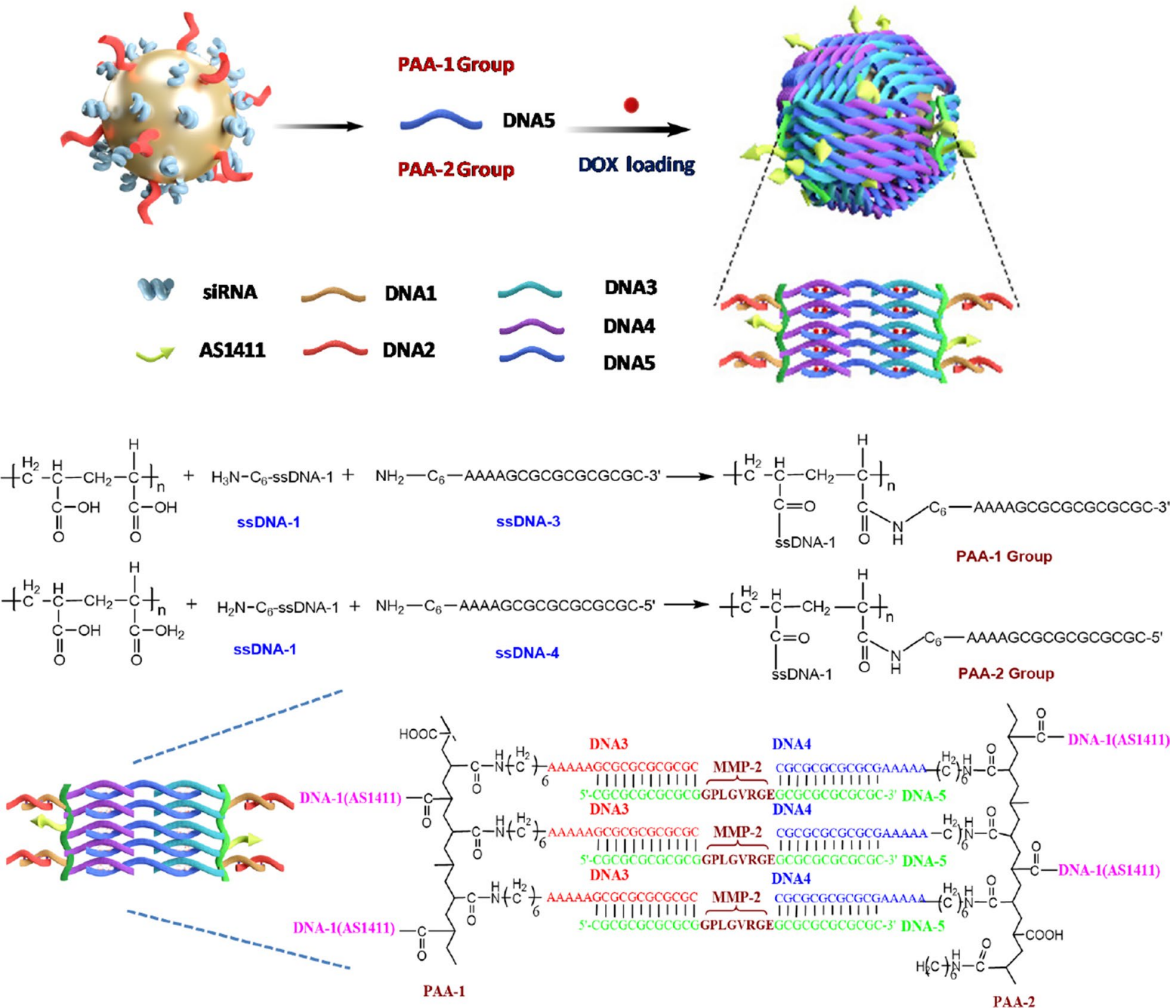
The DNA braiding process and the structure of the Au nanocage

Citrate-coated gold nanoparticles of approximately 10 nm were selected as the core of the nanocage and were synthesized according to a previously reported method [37]. The specific anti-VEGF siRNA duplex with the sequence of 5′-CCCACAUACACACAUAUAUUU-3′ (sense) and 5′-UUGGGUGUAUGUGUGUAUAUA-3′ (antisense) was selected for the subsequent gene therapy, which has been certified to be one of the most efficient siRNA sequences [38]. PAA 2000 was selected for the nanocage construction and was divided into two groups. The first group, PAA-1, was modified with ssDNA-3 (5′-AAAAGCGCGCGCGCGC-3′), ssDNA-1(AS1411 aptamer). The second group, PAA-2, was modified with ssDNA-1(AS1411 aptamer) and ssDNA-4 (5′-CGCGCGCGCGCGAAAA-3′). AS1411 is an aptamer that targets nucleolin, which is abundant in the nucleus of normal cells but is also over expressed in the cell membrane of tumor cells, including prostate, lung, and breast cancers. The modification with AS1411 rendered the Au nanocage capable of delivering therapeutic genes to lung cancer cells. The Au nanoparticles were first modified with thiolated-DNA-2 and thiolated anti-VEGF siRNA through Au-S bonds (DNA-2 : siRNA = 1:10 molar ratio), then reacted with equal molar ratios of modified PAA-1 and PAA-2. The DNA braiding structure on the surface of sphere is shown in Fig. 1.

**Fig. 2:**
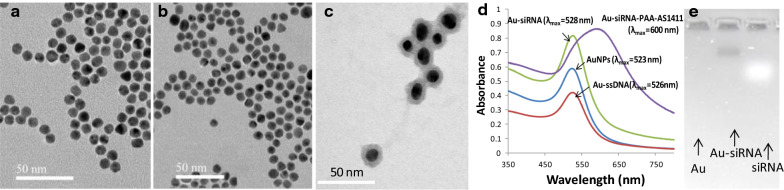
TEM images and UV-vis absorption spectrum of gold nanoparticles. **a** TEM images of ~ 10-nm citrate-coated gold nanoparticles; **b** Low magnification TEM images of Au-siRNA-PAA-AS1411 nanocages; **c** TEM images of Au-siRNA-PAA-AS1411 nanocages with negative staining; **d** Changes in the UV-Vis absorption spectrum of citrate-coated gold nanoparticles, Au-ssDNA and Au-dsDNA; The UV-Vis of the Au-dsDNA and Au-ssDNA (DNA-2 : siRNA = 1:10 molar ratio) exhibited a 5 nm and 3 nm wavelength shift, respectively, compared with the Au nanoparticles, showing the successful modification of DNAs on the surface of gold nanoparticles. **e** The gel image of Au, Au-siRNA, and siRNA showing the successful coating of siRNAs. The band of siRNA located at a molecular weight lower than that of Au-siRNA indicates that siRNAs were successfully conjugated on the surface of Au nanoparticles

To avoid linking multiple gold nanoparticles on same PAA chain, a low concentration of ssDNA-2-modified gold nanoparticle was used. The dynamic light scattering (DLS) (Figure S2) and TEM images (Fig. 2) showed that the Au nanocage were stable and well dispersed. The average diameter of the Au core was approximately ~ 10 nm. The mean particle diameter of the Au nanocage was approximately 16 nm with an approximately 3-nm shell thickness. The UV-Vis of the Au-siRNA and Au modified with ss-DNA exhibited a 5 nm and 3 nm wavelength shift, respectively, compared with the Au nanoparticles, showed that DNAs had successfully been modified on the surface of gold nanoparticles (Fig. 2d).

**Fig. 3:**
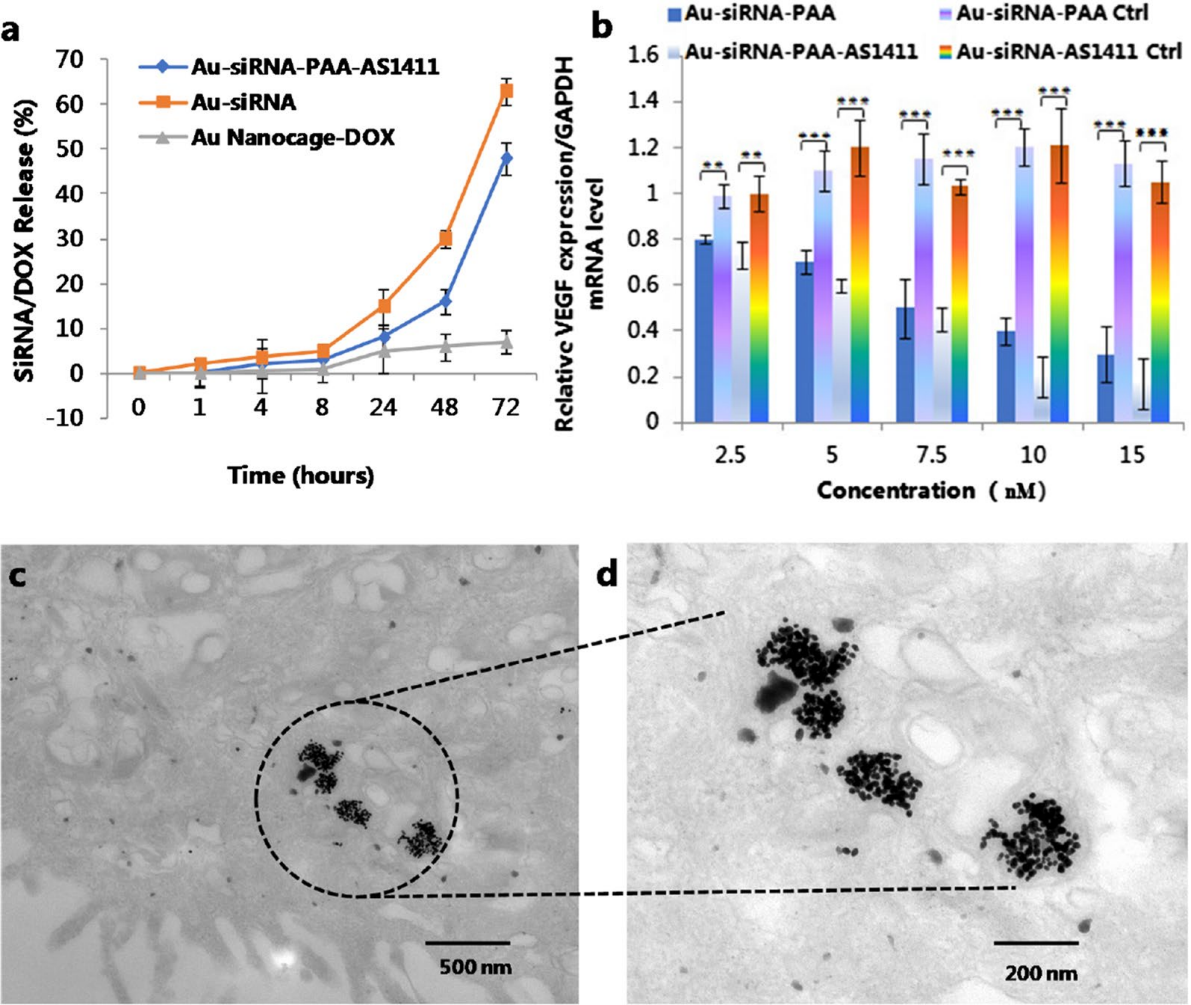
In vitro siRNA / drug release, gene silence efficacy and ultrastructural TEM images of lung tumor tissue from treated mice. **a** siRNA release from the Au-siRNA-PAA-AS1411 nanocages and Au-siRNA; DOX release from Au-siRNA-PAA-AS1411 nanocages; **b** The silencing effect of Au-siRNA-PAA-AS1411 and Au-siRNA-PAA in the lung-adenocarcinoma NCI-H889 cell line. The gene silencing efficacy reached 80% for the Au-siRNA-PAA-AS1411 group with a concentration of 10 nM Au-siRNA-PAA-AS1411 and up to 48 h of incubation (n = 3, ***P < 0.005); **c**, **d** TEM images of NCI-H889 cells incubated with 500 µg mL^− 1^ Au-siRNA-PAA-AS1411 nanocages

To evaluate the stability of the Au nanocages in serum, the in vitro release of siRNAs from the Au nanocages was investigated. In this experiment, siRNAs were modified with Cy5. The Au nanocages were incubated in cell culture medium containing 10% serum for up to 72 h. During the first 8 h, approximately 3% of the siRNAs were released. The cumulative release of the siRNA increased to 8% during the first 24 h. The total release of siRNA reached 16% and 48% in the following 48 and 72 h, respectively. Considering the antitumor efficiency of the Au nanocage, Doxorubicin hydrochloride (DOX) was also loaded into the gold nanocage in the last step of the Au nanocage construction according to the mechanism that DOX can preferentially intercalate into double-stranded -GC- base pairs to form a tightly coupled complex. The DOX release behavior was also investigated in cell culture medium containing 10% serum for up to 72 h. In the first 8 h, the cumulative release of DOX was controlled to within 1%, and then the release increased to 5% at the 24-h time point. The total amount of DOX release was within 7% for up to 72 h incubation, which effectively avoid the drug leakage on the way of distributing to the tumor sites (Fig. 3a). In order to study the release behavior of DOX from the gold nanocage at tumor microenvironment, the DOX release behavior at different pH solution were investigated and were shown in Additional file 1: Figure S3. The cumulative release of DOX was up to 28% at 24-h time point.

### In vitro targeting and anti-tumor effect of gold nanocages

**Fig. 4:**
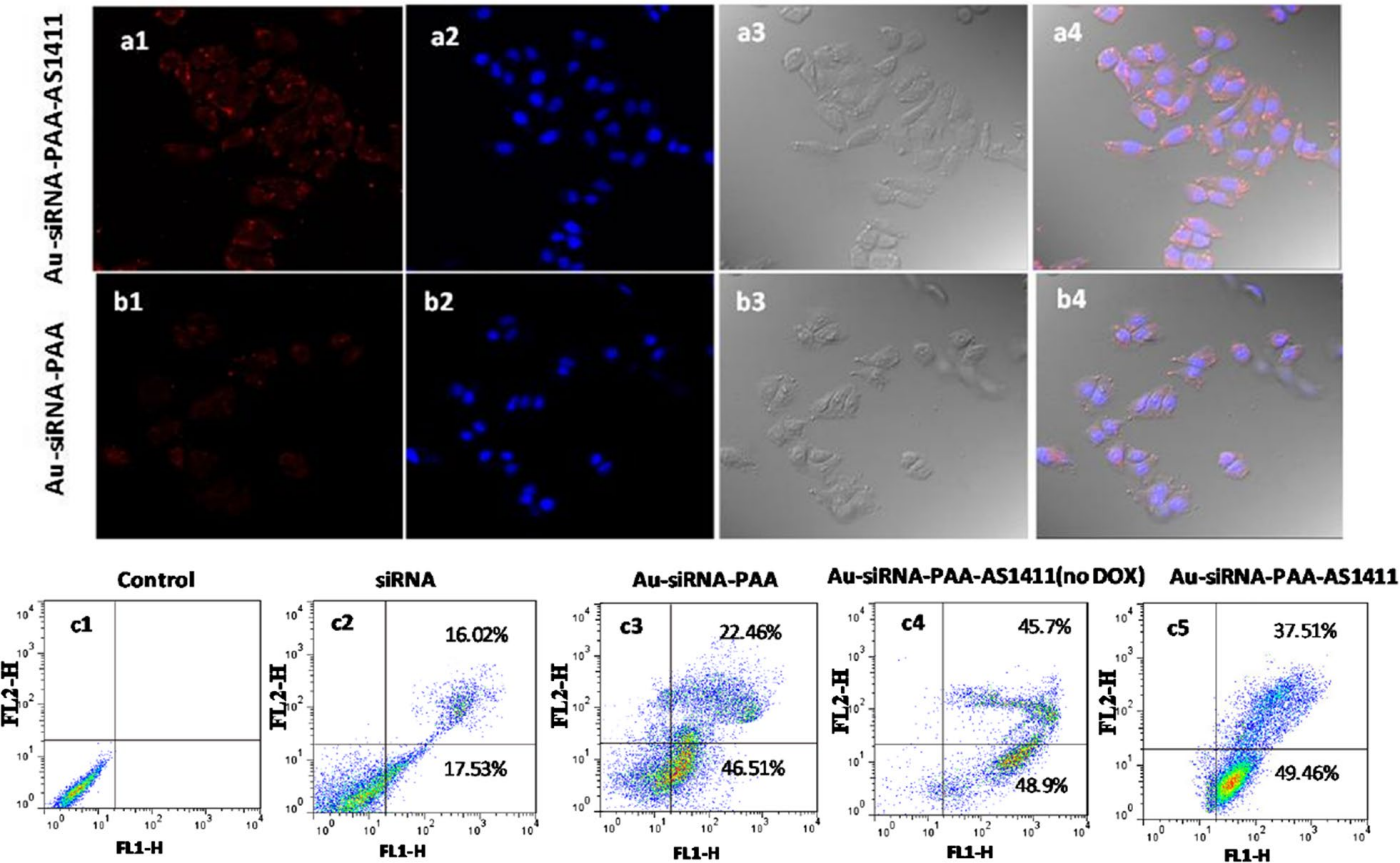
Confocal images and cell apoptosis levels of NCI-H889 cells incubated with the Au-siRNA-PAA-AS1411 /Au-siRNA-PAA nanocages. **a** NCI-H889 cells incubated with 100 µg mL^− 1^ Au-siRNA-PAA-AS1411 for 1 h; **b** NCI-H889 cells incubated with 100 µg mL^− 1^ Au-siRNA-PAA for 1 h. Chanel 1: Fluorescence signal of Au nanocages modified with Cy5; Chanel 2: Hoechst 33,342 signal; Chanel 3: Bright field; Chanel 4: Overlapped field. Magnification: ×40; **c** Flow cytometric analysis of NCI-H889 cell apoptosis induced by siRNA, Au-siRNA-PAA and Au-siRNA-PAA-AS1411 with the MMP-2 enzyme at 37 °C for 24 h

The lung cancer cell line NCI-H889, which was generated from disseminated cancer cells from a KPT mouse, were selected for the in vitro investigation of the cellular uptake and antitumor efficiency of the Au nanocages. The ultrastructural TEM images revealed that the Au-dsDNA-PAA-AS1411 nanoparticles were swallowed by the NCI-H889 cells and were mainly concentrated in the endosomes or lysosomes (Fig. 3c, d). Confocal images of Au nanocages with and without the AS1411 aptamer modification revealed that both Au-siRNA-PAA-AS1411 and Au-siRNA-PAA could enter the NCI-H889 lung cancer cells. The apparent uptake of Au-siRNA-PAA-AS1411 was observed after 1 h of incubation. The uptake of Au-siRNA-PAA was less efficient than that of Au-siRNA-PAA-AS1411 at same incubation time, as evidenced by the weaker fluorescence intensity (Fig. 4a, b). The VEGF silencing efficiency of both Au-siRNA-PAA-AS1411 and Au-siRNA-PAA was evaluated in NCI-H889 lung cancer cells. Both Au-siRNA-PAA-AS1411 and Au-siRNA-PAA exhibited an apparent gene silencing efficiency in a dose-dependent manner. A gene silencing efficacy as high as 80% was observed for the Au-siRNA-PAA-AS1411 group at the mRNA level by qPCR at a dose of 10 nM gold nanocages up to 48 h of incubation (Fig. 3b). The VEGF gene silencing efficiency tends to be stable when the concentration of Au-siRNA-PAA-AS1411 higher than 10 nM. The antitumor efficacy of the Au-siRNA-PAA-AS1411 nanocages was also evaluated by flow cytometry analysis (Fig. 4c). Annexin V-FITC/Propidium Iodide double-staining was used to determine the apoptosis rate of NCI-H889 cells induced by siRNA or siRNA combined with the chemotherapy drug and laser irradiation. The cells were divided into five groups, namely, the control group, the siRNA group, the Au-siRNA-PAA group and the Au-siRNA-PAA-AS1411 group. For the siRNA group, the cells were incubated with siRNA and treated with gene therapy only; for the Au-siRNA-PAA group, the cells were incubated with DOX-loaded Au-siRNA-PAA nanocages and treated with the combination of gene and chemotherapy therapy; for the Au-siRNA-PAA-AS1411 group, the cells were incubated with DOX-loaded Au-siRNA-PAA-AS1411 nanocages and target-treated with gene therapy, chemotherapy and laser irradiation; for the Au-siRNA-PAA-AS1411(no DOX) group, cells were incubated with Au-siRNA-PAA-AS1411 without DOX loading and targeted treated with the combination of siRNA and thermotherapy. The untreated cells served as the control group. Maximal apoptosis and necrosis were observed in the Au-siRNA-PAA-AS1411 group, with subpopulations exhibiting as much as 49.46% early apoptosis and 37.51% late apoptosis. The cell apoptosis level decreased to 68.97% for the Au-siRNA-PAA group; however, both of these groups exhibited much higher cell apoptosis levels compared to the siRNA group.

### In vivo targeting and anti-tumor effect of gold nanocages

**Fig. 5:**
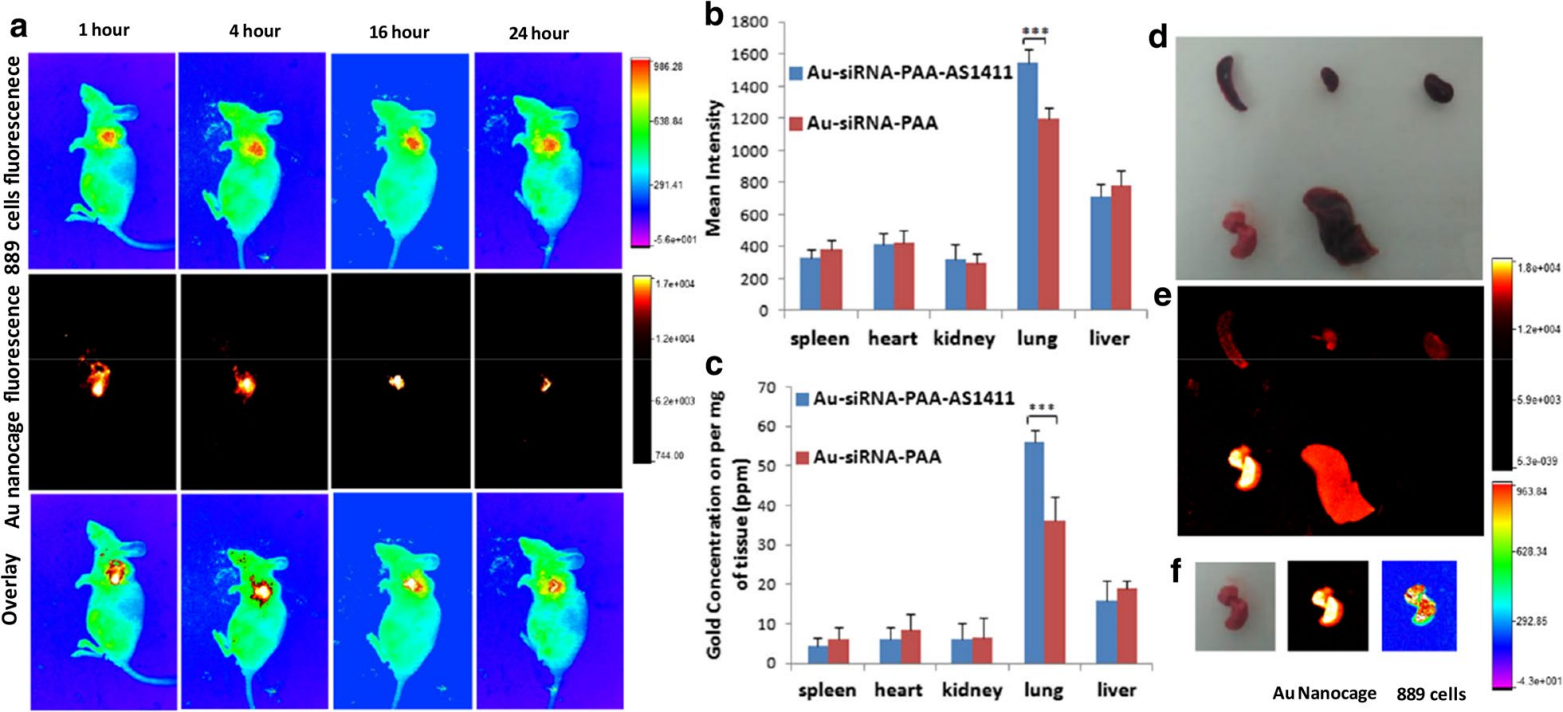
The ability of gold nanocages to deliver anti-VEGF siRNA to the lung cancer orthotopic murine model of BALB/c nude mice expressing NCI-H889 lung cancer cells. **a** Representative noninvasive imagings of NCI-H889 tumor-bearing nude mice in vivo (inhalation-mediated local delivery of 1 mL 800 µg mL^− 1^ Au-siRNA-PAA/Au-siRNA-PAA-AS1411 gold nanocages); **(b)** Quantitative biodistribution of self-assembled nanoparticles in lung tumors and organs based on the ROI (region of interest) analysis of the mean fluorescence intensity of each organ; **c** Quantification of Au accumulated in organs by ICP-MS. The data of Au-siRNA-PAA-AS1411 and Au-siRNA-PAA distribution in liver, spleen, lung (tumor), kidney and heart at 7 days after the inhalation-mediated local delivery of gold nanocages. The data in b and c are expressed as the mean ± SD (n = 6, ***P < 0.005) in each study group; **d** The bright field organ imaging of Au-siRNA-PAA-AS1411-treated mice; **e** The in vitro organ imaging of Au-siRNA-PAA-AS1411-treated mice; **f** Representative NCI-H889 lung-adenocarcinoma cells fluorescence and Au nanocage fluorescence analysis of ex vivo lungs from Au-siRNA-PAA-AS1411-treated mice

Next, Au-siRNA-PAA-AS1411 was investigated for its ability to deliver anti-VEGF siRNA to a lung cancer orthotopic murine model of BALB/c nude mice expressing NCI-H889 lung cancer cells. To avoid the nonspecific uptake of the antitumor drugs, a complex system which allows for the inhalation-mediated local delivery of an anticancer drug was employed to administer the Au-siRNA-PAA/ Au-siRNA-PAA-AS1411 nanocages to the lung tumors. In vivo biodistribution imaging studies were applied for the investigation of the tumor-targeting ability of Au-siRNA-PAA-AS1411. BALB/c nude mice bearing NCI-H889 lung tumors were treated with 1 mL of 800 µg mL^− 1^ Au-siRNA-PAA-AS1411 and Au-siRNA-PAA through inhalation-mediated local delivery. In vivo imaging was performed over a time-course from 1 to 24 h. As shown in Fig. 5, the Au-siRNA-PAA-AS1411 nanocages were randomly concentrated at the lung site at 1 h post-delivery. At the 4-hr time point, the fluorescence signal from the gold nanocages became decreased and more concentrated toward the tumor signal; thereafter, until 16 hrs, the gold nanocage signal exhibited a great degree of overlay with tumor signal. The signal lasted up to 24 h at the lung tumor site. The quantitative biodistribution of Au-siRNA-PAA-AS1411 gold nanocages was also investigated by isolated organ imaging and ROI (region of interest) analysis of the mean organ fluorescence intensity (Fig. 5b, d, e). The results showed that the Au-siRNA-PAA-AS1411 gold nanocages could target the lung tumor, and moreover, they exhibited a long retention time. The fluorescence intensity of the Au-siRNA-PAA-AS1411 gold nanocages on the lung (tumor) was much stronger compared with other organs. A lower mean fluorescence intensity was also observed for the Au-siRNA-PAA group (Fig. 5b). The fluorescence signal from the Au-siRNA-PAA-AS1411 gold nanocages at the lung (tumor) organ exhibited a high degree of overlay with the lung tumor signal (Fig. 5f). Similarly, inductively coupled plasma mass spectrometry (ICP-MS) revealed that after 7 days of treatment with the Au-siRNA-PAA-AS1411 nanocages, both Au-siRNA-PAA and Au-siRNA-PAA-AS1411 almost exclusively accumulated in the lung tissue (Fig. 5c). As much as an ~ 12-fold increase was observed for the Au-siRNA-PAA-AS1411-treated group compared with other organs. The Au-siRNA-PAA group also exhibited an accumulation of the nanoparticle signal, but it was much lower compared with the Au-siRNA-PAA-AS1411 group, with an approximate 6-fold increase compared with other organs. Only minimal accumulation was observed in other organs. These findings indicate that Au-siRNA-PAA-AS1411 targeted and accumulate in the tumor-bearing lung after the inhalation-mediated local delivery, and minimal Au-siRNA-PAA-AS1411 nanocages diffused to other organs from the lung.

### Drug and gene loading gold nanocages inhibited lung cancer growth and prolonged the survival of mice

**Fig. 6:**
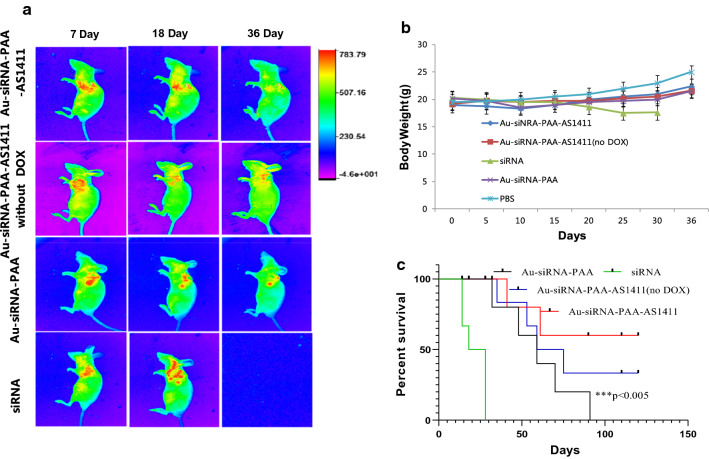
Lung cancer inhibition ratio and the survival rate of the mice. **a** Representative non-invasive imagings of lung-adenocarcinoma NCI-H889 tumor-bearing nude mice treated with siRNA, Au-siRNA-PAA, Au-siRNA-PAA-AS1411(no DOX) and Au-siRNA-PAA-AS1411 nanocages at days 7, 18 and 36; **b** Body weight change curve of tumor-bearing mice over the 36 days of treatment. **c** Kaplan–Meier survival curves of mice treated with siRNA (green), DOX-loaded Au-siRNA-PAA (black), DOX-loaded Au-siRNA-PAA-AS1411 (red) and Au-siRNA-PAA-AS1411 without DOX(blue). Data represent the mean ± standard deviation (n = 6, ***P < 0.005)

Having established that the Au-siRNA-PAA-AS1411 nanocage could selectively deliver siRNA and DOX to the tumor sites, we next evaluated the lung tumor inhibition ratio using NCI-H889 lung tumor-bearing mice treated with Au-siRNA-PAA-AS1411(no DOX), Au-siRNA-PAA and Au-siRNA-PAA-AS1411 loaded with DOX. To create an orthotopic murine model of lung cancer, NCI-H889 lung cancer cells were injected subcutaneously into the lungs of nude mice. Accordingly, BALB/c nude mice bearing NCI-H889 lung tumors were treated with DOX-loaded Au-siRNA-PAA-AS1411 gold nanocages at a dose of 1 mL (800 µg mL^− 1^ ) and administered on days 12, 15, 18, 21, 24, 27, 30, 33 and 36 (Au-siRNA-PAA-AS1411 group). Considering the side effect of the drugs, 808-nm laser irradiation was performed 4 h after the inhalation administration at a power density of 1 W cm^− 2^ for 5 min. Three control groups of mice were treated as follows: (a) the siRNA group, in which mice were administered with siRNA and treated through gene therapy only; (b) the Au-siRNA-PAA group, in which mice were administered with DOX-loaded Au-siRNA-PAA nanocages and passively treated with the combination of siRNA and chemotherapy; and (c) the Au-siRNA-PAA-AS1411(no DOX) group, in which mice were administered with Au-siRNA-PAA-AS1411 without DOX loading and targeted treated with the combination of siRNA and thermotherapy. For the thermotherapy therapy groups, the surface temperature of the lungs was increased to 48 ℃ after the laser irradiation. The tumor inhibition ratio was calculated using a live animal imaging system to monitor the tdTomato-labeled NCI-H889 lung tumor fluorescence (Fig. 6). Compared to the siRNA groups, tumor-bearing mice subjected to targeted treatment with the combination of gene therapy, chemotherapy and photothermal therapy exhibited the best tumor inhibition ratio, with nearly complete tumor regression after 36 days of treatment. More than a three-fold decrease in tdTomato signal was observed after 18 days of treatment compared with the siRNA group. In comparison, the Au-siRNA-PAA and Au-siRNA-PAA-AS1411 (no DOX) groups also exhibited a good tumor inhibition ratio after 18 days of treatment. The total fluorescence intensity of the Au-siRNA-PAA and Au-siRNA-PAA-AS1411 (no DOX) groups were stronger than that of the Au-siRNA-PAA-AS1411 group after 36 days of treatment, which exhibited relative weaker tumor inhibition efficiency. The Au-siRNA-PAA-AS1411, Au-siRNA-PAA-AS1411 (no DOX) and siRNA-siRNA-PAA groups exhibited a stronger tumor inhibition effect when compared with the siRNA group, as no mice survived after 28 days of treatment with passive targeted gene therapy only. Accordingly, the BALB/c nude mice bearing NCI-H889 lung-adenocarcinoma tumors treated with DOX-loaded Au-siRNA-PAA-AS1411 nanocages exhibited the highest survival rate when the VEGF protein was knocked down in combination with chemotherapy and photothermal therapy (Fig. 6c). In summary, these findings indicate that a combination of gene therapy, chemotherapy and photothermal therapy is far more efficient for tumor inhibition compared with only silencing VEGF using gold nanocages via passive targeting.

The safety of all formulations of gold nanocages was evaluated by monitoring the body weight of the mice. As shown in Fig. 6b, a small decrease in body weight occurred during the first 7 days of administration. However, no significant body weight fluctuation was observed in the following days, suggesting that the gold nanocage doses used in the experiments were within the safe range. A steady increase in weight was observed in the Au-siRNA-PAA, Au-siRNA-PAA-AS1411(no DOX) and Au-siRNA-PAA-AS1411 groups, much better than that of the siRNA group, which was treated via passive targeted genetic therapy only. The siRNA group exhibited a sharp decline in body weight after 15 days of treatment, and no mice survived after 36 days of treatment.(Fig. 6c) These findings confirm that the combination of gene therapy, chemotherapy and photothermal therapy is far more efficient than genetic therapy only, and the combination therapy prolonged the lives of the mice and improved their health to some extent.

**Fig. 7:**
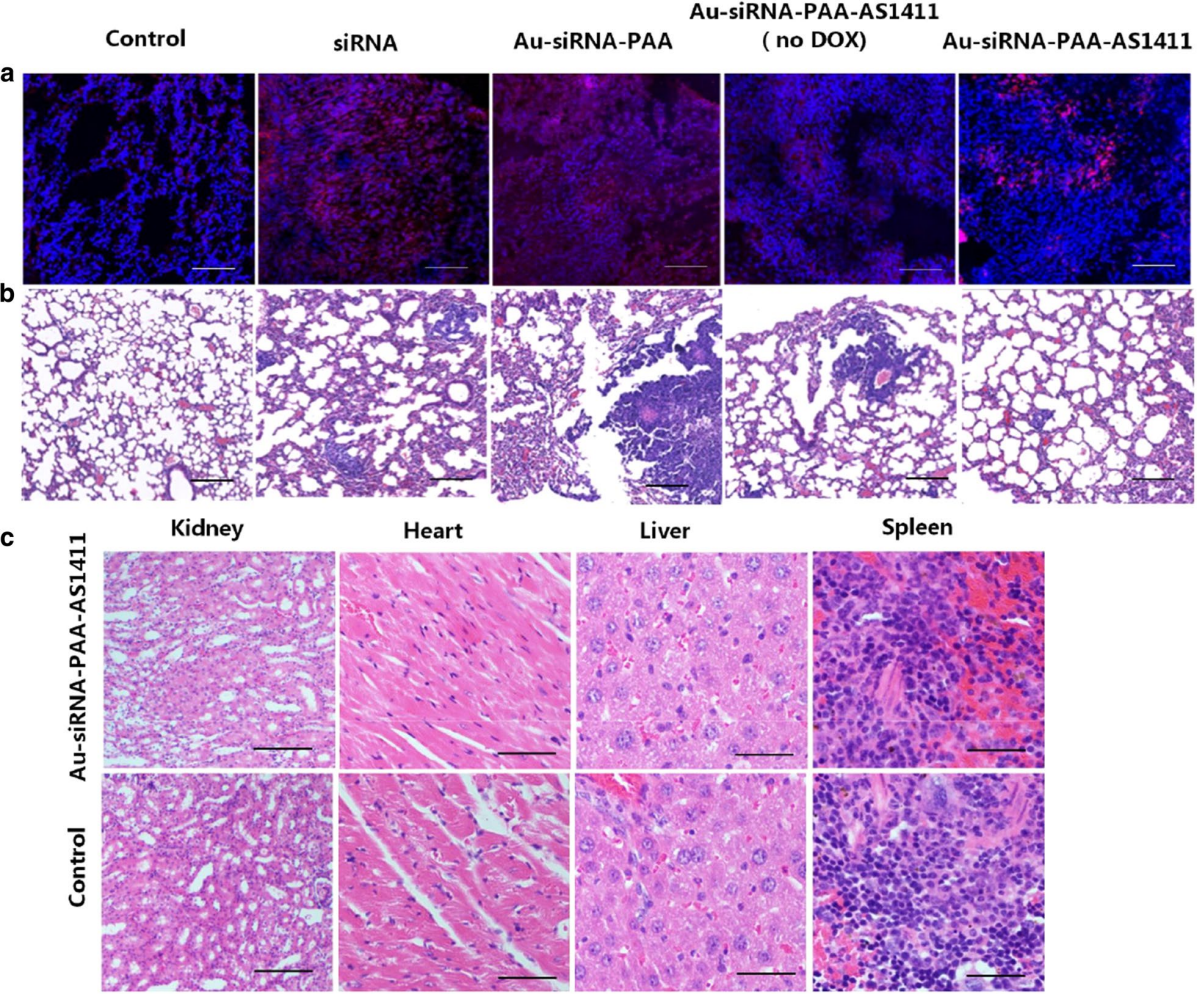
Histology and Immunohistochemistry of mice tissues treated with gold nanocages. **a** Immunohistochemical analysis of VEGF in lung tissue. ~70% reduction of VEGF expression was observed in the lungs treated with Au-siRNA-PAA-AS1411; VEGF (red chanel); Nuclei (blue chanel); **b** Histological analysis of lung tissue treated with Au-siRNA-PAA-AS1411(DOX) compared with the control groups of lung tissue treated with siRNA and DOX-loaded Au-siRNA-PAA; **c** H&E-stained tissue sections from mice subjected to inhalation-mediated local treatment with DOX-loaded Au-siRNA-PAA-AS1411 and PBS for 36 days. Scale bars: 50 µm

The VEGF silencing effect, antitumor efficacy, and the potential toxicity of the gold nanocages were also evaluated by immunohistochemistry and hematoxylin and eosin (H&E) staining (Fig. 7). Immunohistochemical staining for VEGF in the lung (tumor) tissue revealed an ~ 70% reduction in VEGF for the mice treated with Au-siRNA-PAA-AS1411(DOX), which showed a more significant reduction compared to the mice treated with Au-siRNA-PAA-AS1411(no DOX) and Au-siRNA-PAA (Fig. 7a). A clear difference in lung tumor morphology between the siRNA and Au-siRNA-PAA-AS1411 groups was observed in the H&E-stained tissues (Fig. 7b). More tumor foci were seen in the siRNA group. These results indicate that the number of tumor cells was decreased in the mice treated with the DOX-loaded Au-siRNA-PAA-AS1411 nanoparticles through combined multimodal therapy. To further prove the safety of the gold nanocages, tissue sections from Au-siRNA-PAA-AS1411-treated mouse organs were examined to evaluate the potential toxicities. The morphologies of the organs of the treated mice, as shown in Fig. 7c, were similar to those of organs from the untreated healthy mice. Myocardial revealed no myocardial inflammation or necrosis. The liver cells were arranged in order, and no liver cell degeneration, necrosis, or hepatic periportal inflammatory cell infiltration was observed. The glomerulus and tiny tubules of the kidney were normal, with no hyaline change or any necrotic area. Overall, these findings confirmed the good tumor inhibition effect of the combined treatment by Au-siRNA-PAA-AS1411 nanoparticles, and no significant toxicity was evident in mice treated with DOX-loaded Au-siRNA-PAA-AS1411 nanocages.

## Conclusions

Gene therapy is an efficient and promising approach to treat malignant tumors by either degrading mRNAs or inhibiting their translation. To address the problems of the degradation of siRNA in serum, the rapid elimination by renal excretion, and the delivery of sufficient amounts of siRNA to target the tumor site, we developed a new gold nanocage vehicle for the delivery of large amounts of siRNA. To maximize the treatment effect, DOX was loaded into the nanocage and released in a controlled manner at the tumor sites together with the siRNAs. Genetic, chemotherapeutic and photothermal combined targeted treatment was realized with the surface modification with the AS1411 aptamer. To avoid degradation and nonspecific uptake and to maximize the therapeutic effect on lung tumors, the aerosol inhalation method was applied for the in vivo experiments. Our results exhibited that targeted silencing combined with chemo and photothermal therapy can inhibit tumor progression and promote increased survival in mice.

This study is the first attempt to construct a nanocage for siRNA delivery and achieve the controlled release of siRNA and drugs by taking advantage of MMP-2 cleavable peptide. Any kind of siRNA used for lung tumor inhibition could be loaded, and the siRNA sequence could also be replaced with other sequences of siRNA that are more efficient for lung cancer or treating other diseases. In addition, gold nanoparticles exhibited to be an excellent X-ray contrast agent due to their high X-ray attenuation and nontoxicity, which facilitated a more accurate visualization of the controlled target drug release. We expect that this new kind of drug and gene vehicle will be the basis for more efficient therapeutics for treating lung cancer. A similar strategy could be applied to treat other diseases associated with the overexpression of specific markers.

## Materials and methods

### Materials and reagents

The Anti-VEGF antibody was purchased from abcam. DNAs and siRNAs were purchased from Sangon Biotech (Shanghai) and Jinweizhi Company. DOX were purchased from Aladdin Company (Shanghai, China).

### Synthesis and characterization of gold nanoparticles

The 10-nm gold nanoparticles were synthesized by first dissolving HAuCl_4_ (1 mL, 1% w/v) in Nanopure™ H_2_O water (100 mL) with magnetic stirring, heating until reflux, then subsequently adding 1 mL trisodium citrate (3% w/v). Heating was continued for 7 min, at which time the reaction bottle was immediately placed on ice without stirring. The resulting solution of gold nanoparticles was used without further purification for later experiments. Based on the TEM analysis, the average particle size was approximately 10 nm. The concentration of the AuNPs was analyzed by UV-vis absorbance spectroscopy and calculated according to the following formula: A = εlc (A is the absorbance, ε is the molar absorption coefficient, l is the thickness of the liquid layer, and c is the liquid concentration). The ε is 1.37*108 M^− 1^cm^− 1^ for λ = 523 nm of 10 nm Au nanoparticle [39].

### Surface functionalization of AuNPs

The gold nanoparticles used for oligonucleotide conjugation were first treated with bis(p-sulfonatophenyl)phenylphosphine dihydrate dipotassium salt (BSPP), and then 300 nM gold nanoparticles was incubated with 800-fold of thiolated DNA-2 and siRNA (DNA-2:siRNA = 1:10 molar ratio) in 0.5x TBE for more than 12 hrs. Next, 5 M NaCl was added in six increments to bring the final concentration to 300 mM, and aliquots were taken every 2 to 3 hrs. Centrifugation (8,000 rpm for 40 min) was applied to get rid of the salt and free DNA. Finally, the UV-Vis of the final AuNPs was measured to calculate the molar concentration.

### Gold nanocage construction

PAA functionalization with oligonucleotides was performed by carbodiimide chemistry assisted by N-hydroxysuccinimide (EDC/NHS coupling reaction) between the carboxylated PAA and the primary amine groups of the oligonucleotides. PAA-1 was conjugated with 5-fold molar ratios of DNA-1, DNA-3 and the AS1411 aptamer. The resultant products were purified by dialysis for 72 hrs. PAA-2 was conjugated with 5-fold molar ratios of DNA-1, DNA-4 and the AS1411 aptamer. 20 nM DNA-2 oligonucleotide functionalized gold nanoparticles were incubated with 100 nM PAA-1, PAA-2 and 150 nM ssDNA-5 in 0.5x TBE overnight. The final gold nanocages were purified by centrifugation at 10,000×*g* for 30 min in 0.5 × TBE 2 to 3 times. To determine the average size, particle distribution and morphology of the gold nanocages, samples were analyzed through DLS and field emission TEM (JEM-2100F). 1 mg mL^− 1^ uranyl acetate was selected for negative staining. The gold nanocage solution was dripped onto a carbon film-supported copper mesh and allowed to air dry before being subjected to TEM observation.

### DOX loading in the Au-siRNA-PAA-AS1411 nanocage

To investigate the amount of DOX loading in the DNA nanostructure, 5 µM Au-siRNA-PAA-AS1411 nanocage was incubated with DOX (1 mM) in 10 mM PBS (pH 7.4) for 24 hr. Then centrifuge, test the concentration of DOX in the supernatant. The molar ratio of Au-siRNA-PAA-AS1411: DOX was 1:50.5 and this ratio was utilized in the subsequent experiments.

### In vitro siRNA/drug release from the gold nanocages

A dialysis method was employed to investigate the release behavior of siRNA and DOX from the gold nanocages. First, 3 mL of gold nanocage was transferred into a dialysis bag with 18,000 MWCO. The dialysis bag was then dipped into a 50-mL cell culture medium containing 10% serum and incubated at 37 °C with continuous stirring at 200 rpm for as long as 72 h. The, 0.3 mL of the external cell culture medium was removed for analysis at predetermined time intervals, and an equal volume of dialysis solution was replenished. The amount of Cy5 modified siRNA released was quantified by measuring the fluorescence signal of Cy5. The DOX concentration was measured by HPLC.

### Cell culture

The lung-adenocarcinoma NCI-H889 cell line was selected for the in vitro targeting and cell apoptosis experiments. The lung-adenocarcinoma NCI-H889 cell line was cultured in DMEM with 10% heat inactivated fetal bovine serum (Gibco), 100 units of potassium penicillin and 100 µg of streptomycin sulfate per 1 mL of culture media at 37 °C in 5% CO_2_.

### Cellular uptake and distribution of self-assembled nanodrugs

The lung-adenocarcinoma NCI-H889 cells were seeded onto 28-mm glass cover slips for 24 h before being incubated with the gold nanocages. Then, the cells were incubated with 100 µg mL^− 1^ Au-siRNA-PAA-AS1411 /Au-siRNA-PAA nanocages at 37 °C for 1 h, then washed twice with PBS and fixed with a 4% paraformaldehyde solution for 30 min at room temperature. The nuclei were then stained with Hoechst 33,342 (10 µg mL^− 1^) at 37 °C for 15 min, and the slides were rinsed three times with PBS. A confocal laser scanning microscope (CLSM) (Leica TCS SP8, Germany) was used to detect the cellular uptake and distribution of gold nanocages in the NCI-H889 cells. To visualize the cellular distribution of gold nanocages via TEM, NCI-H889 lung-adenocarcinoma cells were incubated with 500 µg mL-1 Au-siRNA-PAA-AS1411 nanocages for up to 48 h and then fixed with glutaraldehyde (2.5%) for more than 1 h. Then, the samples were sent to Shanghai Normal University for TEM testing.

### In vivo targeted delivery of gold nanocages

Lung-adenocarcinoma NCI-H889 cells were selected to construct the lung cancer orthotopic murine model. The lung cancer orthotopic murine model was established by subcutaneously injecting NCI-H889 cells into the lungs of six-week-old BALB/c nude mice after making a 0.5 cm cut under the front leg of mice. Six BALB/c nude mice bearing NCI-H889 lung tumors in each group were injected with 1 mL 800 µg mL^− 1^ Au-siRNA-PAA-AS1411 and Au-siRNA-PAA gold nanoparticles through the inhalation-mediated local delivery method. The fluorescence distribution was monitored at 1 h, 4 h, 16 h and 24 h using a small animal in vivo imaging system with the appropriate wavelengths. The mice used for the in vitro organ imaging were killed 4 h after the gold nanocages were administered. All animal experiment was authorized according to the Shanghai Jiao Tong University Animal Care guidelines.

### Tumor inhibition activity of gold nanocages

BALB/c nude mice bearing the NCI-H889 orthotopic murine model of lung cancer were treated with Au-siRNA-PAA-AS1411 gold nanocages on days 12, 15, 18, 21, 24, 27, 30, 33, and 36. Considering the wastage in the course of inhalation administration, 1 mL 800 µg mL^− 1^ Au-siRNA-PAA-AS1411 gold nanocages were used. Then, 808-nm laser irradiation was performed after 4 h of the inhalation administration at a power density of 1 W cm^− 2^ for 5 min. Three groups of orthotopic lung tumor-bearing mice treated with siRNA, DOX-loaded Au-siRNA-PAA and Au-siRNA-PAA-AS1411 (no DOX) were used as the control. All experiments included six mice per treatment group unless otherwise noted.

### Quantitative PCR

Total RNA from lung-adenocarcinoma NCI-H889 cells and from tissues from tumor-bearing mice was extracted using the RNeasy Plus Mini Kit according to the manufacture’s protocol. qRT-PCR was performed with the SuperScript™ III One-Step RT-PCR System with the Platinum™ Taq DNA Polymerase Kit. The samples were placed in the preheated thermal cycler, which was programmed with the following thermal cycling procedure: 45 ~ 60 °C for 30 min; 2 min at 94 °C; 40 cycles 15 s at 94 °C, 30 s at 55 ~ 66 °C, 1 min at 68 °C; finally, 1 cycle 5 min at 68 °C. GAPDH was used as the reference gene.

### Histology and immunohistochemistry

Freshly removed lung tumor tissues and other organs were rinsed with sterile PBS, frozen with liquid nitrogen and sectioned with a cryostat microtome. For the immunohistochemical analysis, lung (tumor) sections were first fixed with 4% paraformaldehyde for 15 min, then treated with 0.25% TritonX-100 for 10 min and washed with PBS three times. Next, the tissues were blocked in PBST containing 1% BSA for 30 min and then washed with PBS 3 times. Then, the tissue was incubated with an anti-VEGF antibody for 1 h at room temperature and then washed three times with PBS. Then, the tissues were incubated with the secondary antibody (Alexa Fluor 532 goat anti-mouse IgG, 1:250, Invitrogen) for 1 h at room temperature and subsequently washed 3 times with PBS. The nuclei were then stained with Hoechst 33,342 (10 µg mL^− 1^) at 37 °C for 15 min and then rinsed three times with PBS. For H&E staining, the organs were cut into small pieces and fixed with 4% paraformaldehyde, embedded in paraffin, sectioned and stained according to the standard procedures. All tissues were harvest alter 36 days treatment except the siRNA group, which were harvest after 28 days treatment. All slices were examined by CLSM (Leica TCS SP8, Germany).

### Statistical analysis

Data are presented as the mean ± SD unless otherwise indicated. Differences between groups were examined using Student’s t-test. Statistically significant P values were indicated in figures or captions as ***P < 0.005; **P < 0.01; *P < 0.05. All in vivo experiments used six mice per treatment group unless noted otherwise.

## Supplementary information

**Additional file 1: Figure S1.** Quantification the amounts of DNA strands loading on gold nanoparticles. (a) 10 nm gold nanoparticles were coated with ~ 130 strands of DNA per nanoparticle, corresponding to a 1:130 molar ratios; (b) The amount of antisense DNAs binding on the gold nanoparticles with 500:1 incubation molar ratio. **Figure S2.** DLS curve of Au-siRNA PAAAS1411. **Figure S3.** Profile of Dox release from the Au-siRNA-PAA-AS1411 nanocage in PBS buffer at 37 °C in pH 7.4 (bottom) and in pH 5.5 (top). **Figure S4.** Profile of siRNA release from the Au-siRNA-PAA-AS1411 nanocage in PBS buffer at 37 °C in pH 7.4 and in pH 5.5. **Table S1.** The sequence of DNAs.

## Data Availability

All data generated or analyzed during this study are included in this article.

## Abbreviations

DOX: Doxorubicin hydrochloride
VEGF: The vascular endothelial growth factor
NPs: Nanoparticles
siRNA: Small interfering RNA
M-MSN: Magnetic mesoporous silica nanoparticle
siVEGF: VEGF-targeted siRNA
PEI: Polyethylenimine
PAA: Polyacrylic acid
ssDNA: Single strand DNA
MMP: Matrix metalloproteinase enzymes
