# Supplementary material for: Au-siRNA@ aptamer nanocages as a high-efficiency drug and gene delivery system for targeted lung cancer therapy
Source: J Nanobiotechnology. 2021 Feb 24;19:54. doi: 10.1186/s12951-020-00759-3 (PMC7905599; doi:10.1186/s12951-020-00759-3)
Supplement: Supplementary file 1 — Additional file 1: Figure S1. Quantification the amounts of DNA strands loading on gold nanoparticles. (a) 10 nm gold nanoparticles were coated with ~ 130 strands of DNA per nanoparticle, corresponding to a 1:130 molar ratios; (b) The amount of antisense DNAs binding on the gold nanoparticles with 500:1 incubation molar ratio. Figure S2. DLS curve of Au-siRNA PAAAS1411. Figure S3. Profile of Dox release from the Au-siRNA-PAA-AS1411 nanocage in PBS buffer at 37 °C in pH 7.4 (bottom) and in pH 5.5 (top). Figure S4. Profile of siRNA release from the Au-siRNA-PAA-AS1411 nanocage in PBS buffer at 37 °C in pH 7.4 and in pH 5.5. Table S1. The sequence of DNAs. [file 12951_2020_759_MOESM1_ESM.pdf]

Supplementary Materials

Au-siRNA@ Aptamer Nanocages as a High-Efficiency Drug and Gene Delivery System for Targeted Lung Cancer Therapy

Yuming Yang[1,2], Yu Han[1], Qiuyang Sun[3], Jin Cheng[1], Caixia Yue[1,4], Yanlei Liu[1], Jie Song[1,2], Weilin Jin[1,2], Xianting Ding[4], Jesús M. de la Fuente[1,5], Jian Ni[1], Xiaoqiang Wang[3], Daxiang Cui[1,2*]*

[1]Institute of Nano Biomedicine and Engineering, Key Laboratory for Thin Film and Microfabrication Technology of the Ministry of Education, Shanghai Engineering Research Center for Intelligent Diagnosis and Treatment Instrument, Department of Instrument Science & Engineering, School of Electronic Information and Electrical Engineering. Shanghai Jiao Tong University, 800 Dongchuan Road, Shanghai 200240, People's Republic of China

[2]National Center for Translational Medicine, Collaborative Innovational Center for System Biology, Shanghai Jiao Tong University, 800 Dongchuan RD, Shanghai 200240, People's Republic of China

[3] Pediatric neurological disease center, Xinhua hospital, Shanghai Jiaotong University School of Medicine, Number 1665，Kongjiang Road, Shanghai, 200092, People’s Republic of China

[4]School of Biomedical Engineering, Shanghai Jiao Tong University, Shanghai 200240, People's Republic of China

[5]Instituto de Nanociencia de Aragon (INA), Universidad de Zaragoza, Zaragoza, 50018, Spain

Corresponding author： E-mail: Wangxiaoqiang419@163.com; dxcui@sjtu.edu.cn

1. Quantification the amount of ssDNA/siRNA binding on the surface of Au nanoparticles

The amount of thiolated ssDNA binding on the surface of Au nanoparticles were determined by the Quant-iT Oligreen ssDNA Detection Kit. In general, BSPP pretreated gold nanoparticles were incubated 800 fold molar ratios of thiolated oligonucleotides. The supernatant were collected by pipette after centrifugation at 8000 rpm for 40 mins. A standard calibration curve was created using the same oligonucleotides at same solvent conditions to that of the supernatant. Prepare an aqueous working solution of the Quant-iT™ OliGreen® reagent by making a 200-fold dilution of the concentrated DMSO solution in 10 mM Tris-HCl, 1 mM EDTA, pH 7.5 (TE). Dilute the experimental oligonucleotide solution in TE to a fina l volume of 1.0 mL in disposable cuvettes, then mix with 1.0 mL of the aqueous working solution of the Quant-iT™ OliGreen® reagent, incubate 5 minutes at room temperature, protected from light. Measure the fluorescence of the sample using same instrument parameters to that used when making the standard calibration curves. Determine the oligonucleotide concentration of the sample from the standard curve. The complementary strand of DNA hybridization efficiency was also quantified using a similar method, but Cy3 modified antisense DNA were used instead of Oligreen to

eliminate the possibility of thiolated oligonucleotides dropped into the solution in the hybridization process. The amount of siRNAs loading on the surface of gold nanoparticles were estimated using the same method, and all waters used in the experiments are NanopureTM H_2O water.

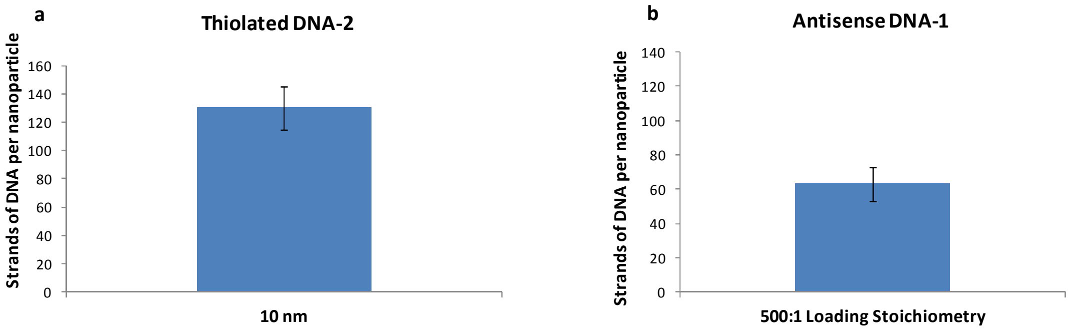

Figure S1. Quantification the amounts of DNA strands loading on gold nanoparticles. (a) 10 nm gold nanoparticles were coated with ~130 strands of DNA per nanoparticle, corresponding to a 1:130 molar ratio; (b) The amount of antisense DNAs bingding on the gold nanoparticles with 500:1 incubation molar ratio.

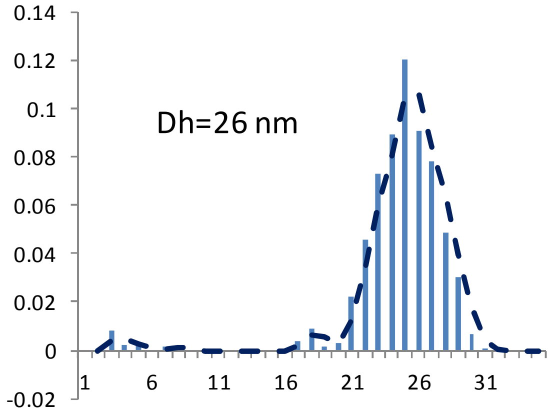

Figure. S2. DLS curve of Au-siRNA-PAA-AS1411 shows the average size of the nanocage is 26 nm. The size measured by DLS is larger than the size measured by TEM, which is due to the fact that the solvation effect and the shrinkage of

nanoparticles in a drying state during TEM sample preparation.

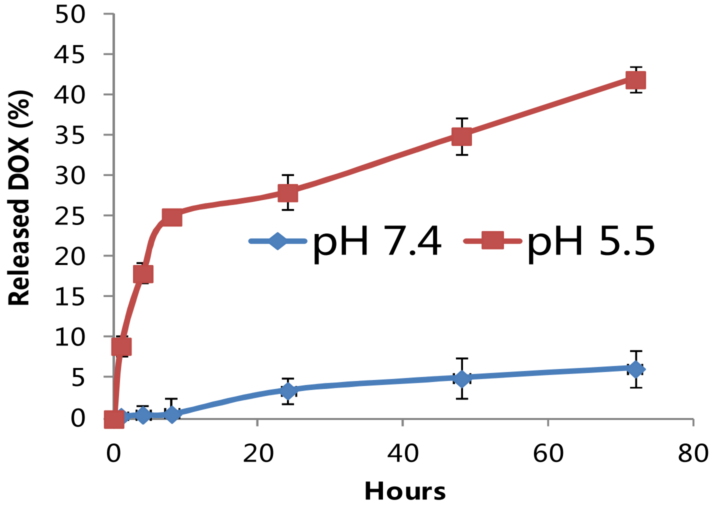

Figure S3. Profile of Dox release from the Au-siRNA-PAA-AS1411 nanocage in PBS buffer at 37°C in pH 7.4 (bottom) and in pH 5.5 (top). Data represent the mean ± standard deviation (n = 3).

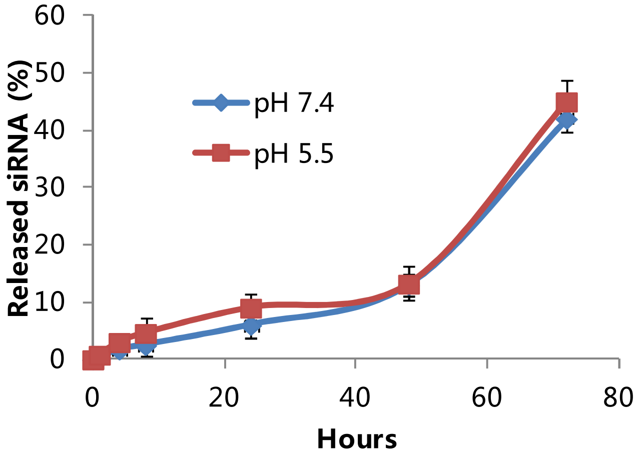

Figure S4. Profile of siRNA release from the Au-siRNA-PAA-AS1411 nanocage in PBS buffer at 37°C in pH 7.4 and in pH 5.5. Data represent the mean ± standard deviation (n = 3).

Table S1: The sequence of DNAs.

DNA Name	Sequence(5'to3')	Purification Method	Modification
**ssDNA-1(AS1411)**	GGTGGTGGTGGTTGTGGTGGTGGTGGTTT	HPLC	5'NH_2-C6-
**ssDNA-2**	AAACCACCACCACCACAACCACCACCACC	HPLC	5'SH-C6-
**ssDNA-3**	AAAAGCGCGCGCGCGC	HPLC	5'NH_2-C6-
**ssDNA-4**	CGCGCGCGCGCGAAAA	HPLC	3'NH_2-C6-
**DNA-5**	CGCGCGCGCGCGGPLGVRGEGCGCGCGCGCGC	HPLC	
